# Early return to work: Single-port vs. multiport laparoscopic surgery for benign ovarian tumor

**DOI:** 10.3389/fsurg.2022.1005898

**Published:** 2022-10-08

**Authors:** Ying Tang, Ming-bo Wen, Bin Su, Hang Wang, Xue-mei Zheng, Ming-tao Yang, Shu Yin, Fan Xu, Hui-quan Hu

**Affiliations:** ^1^ Department of Obstetrics and Gynecology, The Affiliated Nanchong Central Hospital of North Sichuan Medical College, Nanchong, China; ^2^ Department of Obstetrics and Gynecology, Si Chuan Mian Yang 404 Hospital, Mianyang, China

**Keywords:** single-port laparoscopic surgery (SPLS), benign, ovarian tumor, return to work (RTW), surgery

## Abstract

**Objective:**

To compare the return to work (RTW) time between single-port laparoscopic surgery (SPLS) and multiport laparoscopic surgery (MPLS) for benign ovarian tumors.

**Methods:**

A cross-sectional cohort study was conducted, which consisted of 335 women of reproductive age with benign ovarian tumors and who were keen on returning to work as early as possible. Surgical outcomes, postoperative pain score, postoperative satisfaction with the cosmesis score (CS), and the RTW time of the SPLS group were compared with those of the MPLS group. Besides, the RTW time and CS were calculated from the questionnaire survey by a single specialized gynecologist.

**Results:**

Women who met the inclusion criteria were included in the SPLS (*n* = 106) and MPLS groups (*n* = 229). The RTW time in the SPLS group (22.13 ± 27. 06 days) was significantly shorter than that in the MPLS group (46.08 ± 57.86 days) (*P* < 0.001). The multivariate Cox analysis results showed that age (HR = 0.984, 95% CI, 0.971−0.997, *P* = 0.020), SPLS (HR = 3.491, 95% CI, 2.422−5. 032, *P* < 0.001), and return to normal activity time (HR = 0.980, 95% CI, 0.961−0.998, *P* = 0.029) were independent factors of the RTW time.

**Conclusions:**

SPLS may be advantageous in terms of shortening the RTW time for women with benign ovarian tumors.

## Introduction

Benign ovarian tumors are the most common condition in women of reproductive age; of these, 7% are urgent candidates for surgery (1). Single-port laparoscopic surgery (SPLS) has become a common procedure for treating benign ovarian tumors (2). Studies by Capozzi et al. (3) and Yi (4) have shown that single-site surgery seems to be a feasible and safe gynecological procedure for women with benign ovarian tumors. However, Bonollo et al. (5) have reported that current evidence does not seem to demonstrate a clear superiority of laparoendoscopic single-site surgery over multiport laparoscopic surgery (MPLS), except for producing cosmetic results in benign gynecological surgery. Thus, no consensus has been reached on the advantages of SPLS for benign ovarian tumors, including whether it contributes to a shorter return to work (RTW) time (2, 6–8).

Women with benign ovarian tumors, especially single mothers, must return to work as quickly as possible, because their employment income is the main source of family income. Delayed RTW lowers the postoperative quality of life of young women and imposes undue yet substantial costs on society owing to the lost working hours (8). Therefore, it is of great importance to choose a surgical plan that helps women with benign ovarian tumors to reduce their RTW time.

We retrospectively analyzed the data of women with benign ovarian tumors, aiming to explore the potential relationship between the RTW time and SPLS and to identify the potential factors affecting the RTW time of SPLS.

## Materials and methods

### Study population

A cross-sectional cohort study was conducted on 335 women with operative indications for benign ovarian tumors at the Affiliated Nanchong Central Hospital of North Sichuan Medical College between January 2019 and January 2021. According to medical records, 106 women in the SPLS group and 229 women in the MPLS group were analyzed retrospectively. This study was approved by the institutional review board of Nanchong Central Hospital (No. 03053/2019). The need for consent was waived because of data anonymization and the retrospective nature of the study.

Inclusion criteria were patients who received SPLS or MPLS for benign ovarian tumors; who were satisfied with their work and eager to return to work; had a pelvic ultrasound or magnetic resonance imaging to indicate benign ovarian tumors before surgery; had a normal level of tumor markers, including serum carbohydrate antigen 125 (CA125) and human epididymis protein 4 (HE4); were evaluated as according to the American Society of anesthesiologists (ASA) classification I–II; were confirmed to have a benign ovarian tumor by postoperative histopathology.

The exclusion criteria were patients who were unemployed; who received open surgery for benign ovarian tumors; had no intention of returning to work; had severe pain before operation; were obese with a body mass index (BMI) > 30 kg/m^2^ (9); had an obviously malignant tumor on imaging or high serum CA125 levels (500 U/ml) or postoperative histopathology (10); had other concurrent tumors; had severe internal surgical diseases or a history of abdominal surgery.

### Surgical technique

The surgery was performed by two consultant gynecologists who had prior training in performing SPLS. The trial management committee reviewed two unedited laparoscopic videos and patient outcomes to ensure the adequacy of the surgeon's technique (11). All patients underwent the same preoperative preparation and general anesthesia during surgery, with endotracheal intubation and placement in the supine position with the legs slightly separated. Patients underwent surgery with an open Hasson approach for abdominal entry via a single 2 cm vertical umbilical incision. (12). Upon abdominal entry, a multi-instrument access port was used. Single laparoscopic surgery instruments were used for the surgery. The surgical technique for the MPLS procedure conformed to that used by Richards et al. (13). In addition, the pneumoperitoneum was maintained at 12 mmHg. At the end of each surgery, the fascia was identified and closed with a delayed absorbable suture.

### Parameters analyzed

RTW was defined as the time from the date of surgery to the date of time to return to work. The return-to-work questionnaire survey, which included demographics such as age, work status, and specific questions on returning to work, was peer-reviewed and focus-grouped. Participants were requested to they lived far away from our answer the reason for returning to work, job satisfaction ratings and the time to general activity, including sexual intercourse. The participants were suggested regular follow-up. From August 2021 to September 2021, 335 patients completed the questionnaire survey either at our hospital or by telephone because they they lived far away from our hospital.

BMI was categorized by standard criteria by the WHO, while ASA was classified by standard criteria of the American Society of Anesthesiologists’ classification. Data collection included surgical options, histological subtypes, and surgical outcomes. The major surgical outcome was the RTW time; the others were estimated blood loss, specimen retrieval time, operation duration, postoperative pain score, return to normal activity time, postoperative hospitalization, and cosmetic outcomes. The postoperative pain score was assessed using the Numerical Rating Scale (NRS, range: 0–10, 0 = no pain, 10 = unbearable pain) 48 h after surgery. Postoperative cosmesis satisfaction (CS) was evaluated by using the scar satisfaction score (range: 0–10, 0 = not satisfactory, 10 = totally satisfactory). In addition, the questionnaire survey was used to calculate the RTW time and CS.

### Data analysis

Statistical analyses were performed using SPSS software (SPSS 20, Armonk, NY, United States). Quantitative variables with normal distribution were described as mean ± standard deviation. We compared the continuous data by conducting the unpaired Student's *t*-test, while binomial data used Chi-squared tests to show the baseline characteristics of the participants and assess the differences between the two groups. The Chi-square test and Cox regression were adopted to illustrate whether the selected surgical approach affected the RTW time. The Kaplan–Meier method was used to evaluate the value of SPLS in predicting the RTW time for women with benign ovarian tumors. In addition, *P* < 0.05 was defined as statistically significant.

## Results

### Clinicopathological features

A total of 335 patients with benign ovarian tumors were included in this cross-sectional cohort study, out of which 106 (31.64%) were included in the SPLS group and 229 (68.46%) in the MPLS group, according to medical records. There were no differences in terms of age, BMI, ASA rank, surgical options, and histological subtypes between the two groups (*P* > 0.05), and these are summarized in Table 1.

**Table 1:** Clinical characteristics of patients with benign ovarian tumor compared between the SPLS and the MPLS groups.

Varies	SPLS group (*n* = 106)	MPLS group (*n* = 229)	*t* or *χ*^2^	*P*
Age (years)	39.77 ± 13.49	38.35 ± 11.91	0.932	0.353
BMI (kg/m^2^)	28.13 ± 7.12	29.83 ± 7.82	−1.891	0.060
ASA			3.043	0.081
I	56	144		
II	50	85		
Surgical options			0.715	0.398
Ovarian cystectomy	64	127		
Ovariectomy	42	102		
Histological subtype			3.599	0.463
Simple cyst	16	22		
Endometrial cyst of ovary	51	123		
Cystadenoma	3	4		
Teratoma	32	75		
Others	4	5		

BMI, body mass index; ASA, American society of Anesthesiologists; SPLS, single-port laparoscopic surgery; MPLS, multiport laparoscopic surgery.

Cystadenoma was defined as serous and mucinous cystadenoma. Others were defined as the benign ovarian tumor excluding simple cyst, endometrial cyst of ovary, cystadenoma, and teratoma.

### Surgical outcomes

The RTW time was shorter in the SPLS group (22.13 ± 27.06 days) than in the MPLS group (46.08 ± 57.86 days). Specimen retrieval time and the postoperative pain score were shorter in the SPLS group than in the MPLS group (*P *< 0.05, Table 2). The operation duration and CS were higher in the SPLS group than in the MPLS group (*P *< 0.05, Table 2). The mean estimated blood loss in the two groups was similar (*P *> 0.05, Table 2). Moreover, there was no conversion to laparotomy, abdominal wall vascular, nerve injury, urinary system injury, lymphatic leakage, and other complications between the two groups.

**Table 2:** Surgical outcomes of patients with benign ovarian tumor in the SPLS and MPLS groups.

Varies	SPLS group (*n* = 106)	MPLS group (*n* = 229)	Test value	*P*
Estimated blood loss (ml)	51.98 ± 65.16	49.93 ± 51.49	0.311	0.756
Mean dimension of the largest tumor (cm)	5.87 ± 3.00	5.81 ± 2.33	0.185	0.854
Specimen retrieval time (s)	33.93 ± 19.74	47.91 ± 41.05	−4.207	<0.001
Operation duration (min)	86.33 ± 39.71	70.45 ± 27.13	3.735	<0.001
Postoperative pain score (point)	3.10 ± 0.83	5.47 ± 1.08	−19.944	<0.001
Return to normal activity time (hours)	32.31 ± 6.59	36.33 ± 4.27	−5.744	<0.001
Postoperative hospitalization (days)	4.05 ± 0.91	4.50 ± 1.10	−3.725	<0.001
Cosmetic outcomes (point)	4.68 ± 0.98	1.87 ± 1.09	23.676	<0.001
Postoperative time of RTW (days)	22.13 ± 27.06	46.08 ± 57.86	−5.291	<0.001

SPLS, single-port laparoscopic surgery; MPLS, multiport laparoscopic surgery; RTW, return to work.

### Factors influencing return to work time

In the multivariate cox logistic regression, age, ASA, surgical approaches, postoperative pain score, and return to normal activity time were associated with the RTW time (*P* < 0.05, Table 3). Besides, multivariate cox regression analysis showed that age (HR = 0.984, 95% CI, 0.971–0.997, *P* = 0.020), SPLS (HR = 3.491, 95% CI, 2.422–5.032, *P* < 0.001), and return to normal activity time (HR = 0.980, 95% CI, 0.961–0.998, *P* = 0.029) were associated with the RTW time for women with benign ovarian tumors (Table 3).

**Table 3:** Univariate and multivariate Cox proportional hazards analysis of postoperative time of RTW.

Variable	Univariate			Multivariate		
	HR	95% CI	*P*	HR	95% CI	*P*
Age (age)	0.982	0.972–0.993	0.01	0.984	0.971–0.997	0.020
ASA	0.784	0.621–0.989	0.04	0.858	0.626–1.177	0.342
Surgical approach						
SPLS vs. MPLS	3.431	2.650–4.442	<0.001	3.491	2.422–5.032	<0.001
Postoperative pain score (point)	0.756	0.692–0.827	0.004	0.992	0.892–1.103	0.875
Time to return to normal activity (h)	0.965	0.946–0.984	<0.001	0.980	0.961–0.998	0.029

ASA, American Society of Anesthesiologists; SPLS, single-port laparoscopic surgery; MPLS, multiport laparoscopic surgery; RTW, return to work.

### The magnitude of SPLS for return to work time

**Figure 1:**
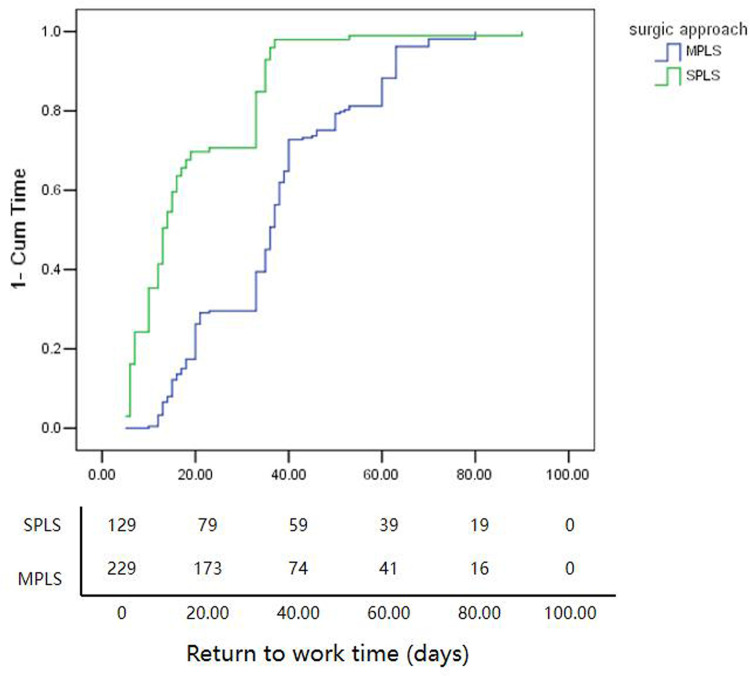
The magnitude of SPLS for RTW. The estimated medium time of RTW in the SPLS group was significantly shorter than those in the MPLS group (18.6 days vs 37.0 days, *P* < 0.001). SPLS, single-port laparoscopic surgery; RTW, return to work time.

In the Kaplan−Meier analysis, the estimated medium time of RTW was significantly shorter in the SPLS group than in the MPLS group (18.6 vs. 37.0 days, *P *< 0.001, as shown in Figure 1).

## Discussion

In this cross-sectional cohort study, we discovered that SPLS was more likely to reduce the RTW time than conventional surgery. Moreover, we showed that SPLS might help reduce specimen retrieval time, gain lower postoperative pain scores, and achieve a superior CS. In addition, our research identified that beyond age and return to normal activity time, SPLS was an independent factor for the RTW time.

Huff et al. (14) showed that return to normal activity time and BMI significantly influenced the time taken for women to work after laparoscopic myomectomy. However, the data on the association between SPLS and RTW after surgery are insufficient. At present, there are no guidelines for the surgeons to chose which one from SPLS and MPLS for resecting benign ovarian tumors. Also, a consensus on the advantages of SPLS in gynecological surgery has not yet been reached. Al-Badawi et al. (15) reported that SPLS was almost feasible and potentially safe, given that no patient required the addition of extra parts or conversion to conventional multiaccess laparoscopy or laparotomy and that patients were satisfied over their reduced postoperative pain. Capozzi et al. (3) reported that a robotic single-port platform seemed feasible and safe for all gynecological surgical procedures. However, higher conversion and complication rates would be considered safe for patients with malignant diseases.

Nevertheless, Schmitt et al. (7) found no significant difference between the two groups in terms of laparotomy conversion rate, postoperative pain, intraoperative blood loss, mean hospitalization, and cosmetic results *via* systematic review and meta-analysis to compare the advantages of SPLS over conventional laparoscopy for surgery. Bonollo et al. (5) also reported that laparoendoscopic single-site surgery does not seem to demonstrate a clear superiority over MPLS, except for better cosmetic results in benign gynecological surgery. Yi (4) reported that single-site incision laparoscopic surgery for adnexal surgery is a safe technique. However, a certain training period for learning the technique is required. In our study, although we did not use a learning curve, we invited the trial management committee to ensure the adequacy of the surgeon's technique. In addition, our results showed the advantages of SPLS in terms of specimen retrieval time, postoperative pain score, cosmetic outcomes, and reduced RTW time in women with benign ovarian tumors.

Admittedly, the majority of patients with benign ovarian tumors are women of reproductive age, especially single mothers with great economic pressure and family responsibilities who are eager to return to work as early as possible. Delayed time to RTW may increase the risks of prolonged sick leave and lower quality of life in postoperative women of reproductive age and lead to undue and substantial costs for society through lost working hours (8). The average RTW time of patients undergoing MPLS in our study was 37 days, meaning most patients had no financial resources during this period. However, the average RTW time in the SPLS group was 18.5 days, which reduced the RTW time to a great extent. Therefore, we can infer that SPLS may reduce the RTW time and be conducive to reducing the economic pressure of family and social burden.

The underlying mechanisms of the benefits of SPLS for benign ovarian tumors have not yet been clarified. However, we attempted to explain the potential mechanisms. First, SPLS might be conducted for postoperative pain relief. Postoperative pain is self-limiting, while the greatest intensity falls on the first and second postoperative days, with much less intensity during the third and fourth postoperative days (16). The probability of shorter pain relief time in the SPLS group is that the likelihood of injury of microvascular nerves and other tissues during surgery is less, with only a slight stimulation to the pain area of the cerebral cortex (17, 18). Second, SPLS conforms to the concept of rapid recovery. As the concept of rapid recovery had successfully demonstrated benefits to surgical patients in the late 20th century, the clinical application of rapid recovery pathways aims at improving surgical outcomes in gynecology (17, 19). SPLS may be conducted to reduce the return to normal activity time and postoperative hospitalization, which is conducive to the rapid recovery of patients and reduces the possibility of cross-infection in hospitals due to long hospitalization days (20). Third, SPLS is more advantageous with regard to specimen retrieval time since its puncture hole is larger than that of MPLS, which provides excellent protection against abdominal wall metastasis and cyst fluid leakage, and also reducing the possibility of metastasis and incision implantation, which might be caused by specimen removal rupture (21). In addition, SPLS maximizes the use of the umbilical folds to cover the scar left by the incision, thus hiding wound healing; a better cosmetic outcome (22), which is more popular with women of reproductive age. Finally, consistent with the results given by Hoyer-Sorensen et al. (23), although the operation duration of SPLS was prolonged in our study due to the “chopsticks effect,” reduced visualization, and the interference between instruments (7), estimated blood loss was minimal and complications such as injury to the nerves and the urinary system did not arise. Therefore, it can be concluded that SPLS is safe and feasible for treating benign ovarian tumors.

However, our study has some limitations. First, the cross-sectional cohort design makes it difficult to state that SPLS is a protective factor for the postoperative time of RTW. However, our study provides details of the clinical strategy for benign ovarian tumors. Second, the potential bias arising out of the influence of the differences of surgical skills on outcomes cannot be completely eliminated; however, we asked the trial management committee to ensure the adequacy of the surgeon's technique. Third, vaginal natural orifice transluminal endoscopic surgery (V-NOTES) is an alternative laparoscopic technique that provides single surgical access without leaving abdominal scars. We did not compare the clinical effects between SPLS and V-NOTES. However, in the future, we plan to conduct another study to compare the time to return to work between V-NOTES and SPLS. The other limitation is that the primary outcome might be susceptible to bias because the RTW time was self-reported by women (8), but data were collected on time. However, we believe that the advantages of SPLS will be of interest to clinicians when devising clinical strategies.

## Conclusion

In summary, our preliminary results showed that beyond age and return to normal activity time, SPLS was conducive to a shorter RTW time in benign ovarian tumors, which meant that it was advantageous in terms of rapid recovery after surgery.

## Data Availability

The raw data supporting the conclusions of this article will be made available by the authors, without undue reservation.
